# Supplementary material for: Content-rich biological network constructed by mining PubMed abstracts
Source: BMC Bioinformatics. 2004 Oct 8;5:147. doi: 10.1186/1471-2105-5-147 (PMC528731; doi:10.1186/1471-2105-5-147)
Supplement: Additional File 5 — The original Chilibot query results of the term "long-term potentiation (LTP)" and 22 other terms, limiting the latest references analyzed to the years 1990, 1995, 2000, and 2004. [file 1471-2105-5-147-S5.bz2 › chilibotAdditionalFile5/ltp2004/html/NMDA_ARC.html]

 


 **NMDA** and **ARC** 
  
Found 28 abstracts in PubMed,  **28 abstracts were retrieved and analyzed**.  


---

 Search Google  |
 PDF files only 
|  EDU domain only 

---

**Interactive relationship** (e.g. stimulation, inhibition, etc)

- Repeated MAP administration induces immediate early gene such as c fos, c jun and  **arc** , and the increase in  **arc**  is inhibited by D1 and  **NMDA**  antagonists, suggesting an important role of such genes in inducing reverse tolerance.  Ref: 11915303 Nihon Arukoru Yakubutsu Igakkai Zasshi, 2002
- The rank order of potency for reduction of  **NMDA**  receptor single channel conductance by polyamines was  **ARC**  greater than SP greater than SD greater than PUT = DET, a rank order similar to that for the inhibitory actions of polyamines in receptor binding assays and for the effects of the antagonists on  **NMDA**  receptor whole cell currents.  Ref: 1378923 Mol Pharmacol, 1992
- Selective targeting of newly synthesized  **Arc**  mRNA to active synapses requires  **NMDA**  receptor activation.  Ref: 11343657 Neuron, 2001
- These results demonstrate that  **NMDA**  receptor activation is required for the targeting of  **Arc**  mRNA to active synapses.  Ref: 11343657 Neuron, 2001
- The present results suggest that  **Arc**  may play an important role in the neuronal plasticity through  **NMDA**  activation following focal cerebral ischemia.  Ref: 10501563 Neuroreport, 1999
- Groups of neonatal postnatal day PD 2 pups were injected with MSG, then stimulated on subsequent days PD 3 29 with  **NMDA** , known to induce c fos expression in  **ARC** .  Ref: 9298238 Brain Res Dev Brain Res, 1997
- The ability of  **NMDA**  to enhance LH release was tested in male rats following infusion into the medial preoptic nucleus MPO, anterior hypothalamic nucleus AHY, ventromedial hypothalamic nucleus VMH, and arcuate nucleus  **ARC** .  Ref: 3062296 Life Sci, 1988
- Quantitative reverse transcriptase PCR revealed that messenger RNA levels for the major  **NMDA**  receptor subunit NMDAR1 were significantly lower in the preoptic area and  **ARC**  ME of the middle aged rat on proestrus afternoon.  Ref: 8641183 Endocrinology, 1996
- Quantitative examination of the cellular expression in the ventromedial and arcuate nuclei of the hypothalamus VMH and  **Arc**  showed that 52.5% and 61.5%, respectively, of the neurons endowed with ERalpha mRNA also contain  **NMDA**  2D mRNA.  Ref: 12117550 Brain Res Mol Brain Res, 2002

**Parallel relationship** (e.g. studied together, co-existance, homology, etc.)

- The polyamine antagonists  **ARC** , DET, and PUT produced a voltage dependent reduction in  **NMDA**  receptor whole cell currents and reductions in single channel conductance and average open time, even in the absence of polyamine agonists.  Ref: 1378923 Mol Pharmacol, 1992
- In contrast, a maximal sub lethal dose of  **NMDA**  which produces a different pattern of  **ARC**  FLI, without edema, has no effect on puberty.  Ref: 9466718 Brain Res Dev Brain Res, 1997
- Arcuate nucleus median eminence  **ARC**  ME fragments were isolated from young 2 month old and middle aged 9 to 11 month old rats at 1700 h on proestrus and incubated in vitro with or without the specific glutamate agonists, D, L alpha amino 3 hydroxy 5 methyl 4 isoxazole propionic acid 1 mM, kainate 1 mM, and N methyl D aspartate  **NMDA** .  Ref: 8641183 Endocrinology, 1996
- The discharge patterns of the neurons may be dependent on many factors, including input from the different barosensitive receptor subtypes, the contribution of different ionotropic glutamate receptors  **NMDA**  N methyl D aspartate versus nonNMDA receptors in transmission of the input, effects of different neuropeptide neurotransmitters neuromodulators on afferent transmission, or the order of the neuron within the barosensitive reflex  **arc** .  Ref: 11458673 Ann N Y Acad Sci, 2001
- Plasma LH levels were not affected following microinfusion of  **NMDA**  50 pmole into the AHY, VMH, and  **ARC** .  Ref: 3062296 Life Sci, 1988
- Pups treated neonatally with saline PD 2 showed a robust, age specific expression of FLI in the  **ARC**  following  **NMDA**  treatment.  Ref: 9298238 Brain Res Dev Brain Res, 1997
- The newly synthesized  **Arc**  mRNA localizes selectively at synapses that have experienced particular patterns of activity, and this targeting depends on  **NMDA**  receptor activation.  Ref: 12559831 Neurobiol Learn Mem, 2002
- When micropipette electrodes containing  **NMDA**  receptor antagonists MK801 or APV were positioned in the dentate gyrus during the stimulation period, newly synthesized  **Arc**  mRNA was transported into dendrites but did not localize in the activated lamina.  Ref: 11343657 Neuron, 2001
- We have examined this ability of the medial basal hypothalamus MBH to recover from an excitotoxic insult using the immediate early gene c fos as a developmental marker of  **ARC**  response to glutamate receptor stimulation with N methyl D aspartate  **NMDA** .  Ref: 9298238 Brain Res Dev Brain Res, 1997
- **Arc**  mRNA dynamics return to sender the  **NMDA**  receptor provides the targeting address for  **Arc**  mRNA.  Ref: 11672783 Trends Neurosci, 2001
- The polyamine site modulators, spermine SPM and spermidine SPD, produce dose related biphasic modulation of  **NMDA**  channel currents while another polyamine, arcaine  **ARC** , produces only negative modulatory effects.  Ref: 9754803 Neurosci Lett, 1998
- We conclude that  **NMDA** ... receptors in the CVLM play an important role in the reflex  **arc**  that dilates the airways when hindlimb afferents are stimulated by either muscular contraction or electrical stimulation.  Ref: 7836189 J Appl Physiol, 1994
